# A Preliminary Study on Machine Learning-Based Evaluation of Static and Dynamic FET-PET for the Detection of Pseudoprogression in Patients with IDH-Wildtype Glioblastoma

**DOI:** 10.3390/cancers12113080

**Published:** 2020-10-22

**Authors:** Sied Kebir, Teresa Schmidt, Matthias Weber, Lazaros Lazaridis, Norbert Galldiks, Karl-Josef Langen, Christoph Kleinschnitz, Elke Hattingen, Ulrich Herrlinger, Philipp Lohmann, Martin Glas

**Affiliations:** 1 Department of Neurology, Division of Clinical Neurooncology, University Hospital Essen, University Duisburg-Essen, D-45147 Essen, Germany; sied.kebir@uk-essen.de (S.K.); teresa.schmidt@uk-essen.de (T.S.); lazaros.lazaridis@uk-essen.de (L.L.); 2 DKFZ-Division Translational Neurooncology at the WTZ, DKTK partner site, University Hospital Essen, D-45147 Essen, Germany; 3 German Cancer Research Center (DKFZ), D-69120 Heidelberg, Germany; 4 German Cancer Consortium (DKTK), D-69120 Heidelberg, Germany; 5 Division of Clinical Neurooncology, Department of Neurology, University Hospital Bonn, D-53127 Bonn, Germany; maatthiasweber@gmail.com (M.W.); Ulrich.Herrlinger@ukbonn.de (U.H.); 6 Department of Neurology, Faculty of Medicine and University Hospital Cologne, University of Cologne, D-50937 Cologne, Germany; n.galldiks@fz-juelich.de; 7 Institute of Neuroscience and Medicine (INM-3,-4), Research Center Juelich, D-52428 Juelich, Germany; k.j.langen@fz-juelich.de (K.-J.L.); p.lohmann@fz-juelich.de (P.L.); 8 Center of Integrated Oncology (CIO), Universities of Aachen, Bonn, Cologne, and Duesseldorf, D-50937 Cologne, Germany; 9 Department of Nuclear Medicine, RWTH Aachen University Hospital, D-52074 Aachen, Germany; 10 Department of Neurology, University Hospital Essen, University Duisburg-Essen, D-45147 Essen, Germany; christoph.kleinschnitz@uk-essen.de; 11 Institute of Neuroradiology, University Hospital Frankfurt, D-60528 Frankfurt, Germany; elke.hattingen@kgu.de

**Keywords:** artificial intelligence, amino acid PET, treatment-related changes, tumor progression, glioma

## Abstract

**Simple Summary:**

Pseudoprogression detection in glioblastoma patients remains a challenging task. Although pseudoprogression has only a moderate prevalence of 10–30% following first-line treatment of glioblastoma patients, it bears critical implications for affected patients. Non-invasive techniques, such as amino acid PET imaging using the tracer O-(2-[^18^F]-fluoroethyl)-L-tyrosine (FET), expose features that have been shown to provide useful information to distinguish tumor progression from pseudoprogression. The usefulness of FET-PET in IDH-wildtype glioblastoma exclusively, however, has not been investigated so far. Recently, machine learning (ML) algorithms have been shown to offer great potential particularly when multiparametric data is available. In this preliminary study, a Linear Discriminant Analysis-based ML algorithm was deployed in a cohort of newly diagnosed IDH-wildtype glioblastoma patients (*n* = 44) and demonstrated a significantly better diagnostic performance than conventional ROC analysis. This preliminary study is the first to assess the performance of ML in FET-PET for diagnosing pseudoprogression exclusively in IDH-wildtype glioblastoma and demonstrates its potential.

**Abstract:**

Pseudoprogression (PSP) detection in glioblastoma remains challenging and has important clinical implications. We investigated the potential of machine learning (ML) in improving the performance of PET using O-(2-[^18^F]-fluoroethyl)-L-tyrosine (FET) for differentiation of tumor progression from PSP in IDH-wildtype glioblastoma. We retrospectively evaluated the PET data of patients with newly diagnosed IDH-wildtype glioblastoma following chemoradiation. Contrast-enhanced MRI suspected PSP/TP and all patients underwent subsequently an additional dynamic FET-PET scan. The modified Response Assessment in Neuro-Oncology (RANO) criteria served to diagnose PSP. We trained a Linear Discriminant Analysis (LDA)-based classifier using FET-PET derived features on a hold-out validation set. The results of the ML model were compared with a conventional FET-PET analysis using the receiver-operating-characteristic (ROC) curve. Of the 44 patients included in this preliminary study, 14 patients were diagnosed with PSP. The mean (TBRmean) and maximum tumor-to-brain ratios (TBRmax) were significantly higher in the TP group as compared to the PSP group (*p* = 0.014 and *p* = 0.033, respectively). The area under the ROC curve (AUC) for TBRmax and TBRmean was 0.68 and 0.74, respectively. Using the LDA-based algorithm, the AUC (0.93) was significantly higher than the AUC for TBRmax. This preliminary study shows that in IDH-wildtype glioblastoma, ML-based PSP detection leads to better diagnostic performance.

## 1. Introduction

Despite therapeutic options consisting of maximal safe surgical resection, radiotherapy, and chemotherapy, glioblastoma has a poor prognosis with a median overall survival, depending upon age and O6-methylguanine-DNA methyltransferase (MGMT) promoter methylation status, ranging between 8.3 and 48.1 months [1,2,3,4]. The standard imaging modality for the response assessment is contrast-enhanced magnetic resonance imaging (MRI) [5]. As there is only a small number of established, tumor-directed treatment options in glioblastoma, it is pivotal that effective treatment is not terminated prematurely. One primary reason for the premature cessation of a putatively effective treatment following chemoradiation is a phenomenon called pseudoprogression (PSP).

PSP is defined as a treatment-related tissue reaction that manifests itself as a progressive enhancing lesion on MRI (henceforth referred to as index MRI) within the radiation field that remains stable or ultimately regresses during a further follow-up MRI without further treatment or any change of treatment [6]. While PSP is typically observed within a time frame of 12 weeks following cessation of chemoradiation, it may also occur considerably later [7]. The incidence varies according to different studies between 10 and 30% [8,9]. The unspecific changes in contrast-enhanced (CE) MRI may be associated with a breakdown of the blood-brain barrier due to inflammation, edema, necrosis, or ischemia [9,10,11]. The diagnosis of PSP, as per the modified Response Assessment in Neuro-Oncology (RANO) criteria [12,13], is based on either pathohistological confirmation through repeat surgery or a follow-up MRI (confirmatory MRI) obtained at least 4–6 weeks after the index MRI after chemoradiation completion [12,13]. On the confirmatory MRI, retrospective diagnosis of PSP is made when the progressive enhancing lesions regress or remain stable in size without any change in treatment. Consequently, the diagnosis of PSP can only be made retrospectively when relying on standard MRI. 

To avoid the continuation of ineffective tumor-directed treatment and the discontinuation of efficient treatment, there is a need for timely detection of PSP. MRI perfusion-weighted imaging is an advanced imaging technique to evaluate cerebral blood volume and vascular permeability. The reported metrics for Dynamic Susceptibility Contrast (DSC) and Dynamic Contrast-Enhanced (DCE) MRI perfusion imaging detect PSP with high specificity and sensitivity in the range of 87–92% and 85–86%, respectively [14]. Positron emission tomography (PET) has increasingly gained attention in glioma treatment monitoring. In the last years, several studies have demonstrated that amino acid tracers such as O-(2-^18^F-fluoroethyl)-l-tyrosine (FET), ^11^C-methyl-l-methionine (MET), and 3,4-dihydroxy-6-^18^F-fluoro-L-Phenylalanine (FDOPA) are considerably more sensitive than the most widely used PET (^18^F-fluorodeoxyglucose) tracer in oncology. The analysis of static FET-PET parameters such as the mean and maximum tumor-to-brain ratios (TBR_mean_, TBR_max_) and dynamic FET-PET parameters such as the time-to-peak (TTP), investigated separately or in conjunction, yield high diagnostic accuracies represented by the area under the receiver-operating-characteristic (ROC) curves (AUC) for PSP/TP detection in the range of 79–94% [7,10,15]. A recent study indicated, that the accuracy of pseudoprogression detection using FET-PET may vary by the isocitrate dehydrogenase (IDH) mutation status. However, the investigated cohort was quite heterogenous with included patients with varying glioma histology (glioma II-IV) [16]. Given IDH has emerged as a hallmark gene in glioblastoma diagnostics with a significant impact on tumor metabolism, it is necessary to investigate glioblastoma cohorts with a homogenous IDH mutation status [17].

The advent of artificial intelligence and machine learning techniques had an impact on image-based research [18]. Machine learning has the potential to enhance the output from image-based data significantly and has been shown useful in many cases [19,20]. In this retrospective analysis, we attempted to investigate whether machine learning techniques may improve the FET-PET-based discrimination between PSP and true progression (TP) in patients with newly diagnosed isocitrate dehydrogenase (IDH) wildtype glioblastoma.

## 2. Methods

### 2.1. Study Design

In this preliminary study, we retrospectively screened the institutional database of the Division of Clinical Neurooncology at the University of Bonn Medical Center and University Hospital of Cologne for patients meeting the following criteria:

- Patients with a neuropathological diagnosis of an IDH-wildtype glioblastoma;
- Completed radiotherapy plus concomitant chemotherapy with either temozolomide (TMZ) or temozolomide/lomustine (TMZ/CCNU);
- CE-MRI suspicious for tumor progression (index MRI) according to the modified RANO criteria [13,21], while under or after first-line treatment with adjuvant alkylating chemotherapy with TMZ or TMZ/CCNU;
- Dynamic FET-PET performed shortly after CE-MRI;
- Confirmation of PSP or TP by histopathology or confirmatory MRI.

There was no treatment change between index MRI and confirmatory MRI. The latter was obtained no earlier than four weeks after index MRI.

All patients gave written informed consent to have their data published anonymously. The local ethics committee approved this retrospective preliminary study (Ethik-Kommission Universität Duisburg-Essen, Code of ethics approval: 20-9431-BO). The IDH mutation status was determined by immunohistochemistry with an IDH-R132H-specific antibody. In patients younger than 55 years at diagnosis, there is a non-negligible probability (roughly 12%) that IDH mutations are not captured by immunohistochemistry directed against IDH-R132H [22]. Therefore, patients younger than 55 years at diagnosis, in whom immunohistochemistry yielded a negative result, DNA sequencing of IDH1 codon 132 and IDH2 codon 172 was conducted [23,24].

### 2.2. PET Imaging with FET

As described previously, the amino acid FET was produced through nucleophilic ^18^F-fluorination with a radiochemical purity of greater than 98%, specific radioactivity greater than 200 GBq/µmol, and a radiochemical yield of about 60% [25]. According to national and international guidelines for brain tumor imaging using labeled amino acid analogues [26,27], all patients fasted for at least 4 h before the PET measurements. All patients underwent a dynamic PET scan from 0 to 50 min post-injection of 3 MBq of FET per kg of body weight at the department of Nuclear Medicine of the RWTH Aachen University Hospital at the Research Center Juelich. PET imaging was performed either on an ECAT Exact HR+ PET scanner in 3-dimensional mode (Siemens, Erlangen, Germany; axial field of view, 15.5 cm) or simultaneously with 3 T MR imaging using a BrainPET insert (Siemens, Erlangen, Germany). The BrainPET is a compact cylinder that fits into the bore of the Magnetom Trio MR scanner (axial field of view, 19.2 cm) [27]. Iterative reconstruction parameters were 16 subsets, six iterations using the ordered subset expectation maximization (OSEM) algorithm for the ECAT HR+ PET scanner and two subsets, 32 iterations using the OP-OSEM algorithm for the BrainPET. Data were corrected for random, scattered coincidences, dead time, and motion for both systems. Attenuation correction for the ECAT HR+ PET was based on a transmission scan, and for the BrainPET on a template-based approach [27]. The reconstructed dynamic data set consisted of 16 time frames (5 × 1 min; 5 × 3 min; 6 × 5 min) for both scanners. To account for the physical differences of the two PET scanners concerning the spatial resolution as well as reconstruction parameters, correction methods, and post-processing steps, a 2.5 mm 3D Gaussian filter was applied to the BrainPET data before further processing, resulting in an image resolution of approximately 4 mm (image resolution of the ECAT HR+ PET scanner, around 5 mm). In phantom experiments using spheres of different sizes to simulate lesions, this filter kernel demonstrated the best comparability between PET data obtained from the ECAT HR+ PET and the BrainPET scanner [17].

### 2.3. FET-PET Data Analysis

Uptake of FET in the tissue was expressed as a standardized uptake value (SUV) by dividing the radioactivity (kBq/mL) in the tissue by the radioactivity injected per gram of body weight. For the evaluation of FET-PET data, summed PET images from 20–40 min post-injection were used. A large spherical volume-of-interest (VOI) (30 mm diameter, 14 mL volume) was positioned in the contralateral unaffected hemisphere including white and grey matter [28]. The tumor volume was determined by a 3-dimensional auto-contouring process using a tumor-to-brain ratio (TBR) of 1.6 or more [29,30]. TBRmean was calculated by dividing the mean SUV of the tumor VOI by the mean SUV of healthy brain tissue. For the calculation of the maximal amino acid uptake, a spherical VOI (16 mm diameter, 2 mL volume) was centered on the voxel with the maximum tumor uptake [31]. TBRmax was calculated by dividing the mean SUV of the 16 mm diameter VOI by the mean SUV of healthy brain tissue. Time-activity curves (TAC) were generated by applying the tumor and brain VOIs to the entire dynamic data set. For TAC evaluation, the TTP; time in minutes from the beginning of the dynamic acquisition up to the maximum SUV of the lesion was determined. In cases with steadily increasing FET uptake without identifiable peak uptake, the end of the dynamic PET acquisition was defined as TTP.

### 2.4. Definition of MRI Acquisition Time Points

In this preliminary study, MRI data at three different acquisition time points were considered. The index MRI was defined as the MRI at initial suspicion for tumor progression or pseudoprogression according to the modified RANO criteria. The baseline MRI was acquired before the index MRI and is compared against it to indicate whether criteria for tumor progression or pseudoprogression have been met. The follow-up MRI was acquired after the index MRI and is used to confirm or reject pseudoprogression in the event that histopathological validation of the diagnosis was not available.

### 2.5. Diagnosis of True Progression/Pseudoprogression

The diagnosis of TP was made when progressive contrast-enhancing lesions, according to the modified RANO criteria [12,13], were noted on index MRI and when the further progression of contrast enhancement was found in a confirmatory MRI at least four weeks later. By contrast, the diagnosis of PSP was applicable when the confirmatory MRI showed stabilization or regression of the contrast-enhancing lesions. Prerequisite for both diagnoses was the lack of changes in the treatment regimen between index and confirmatory MRI.

### 2.6. Machine Learning Algorithm

We devised a machine learning algorithm that differs from how FET-PET data is conventionally approached in a binary classification problem. With conventional analysis, the analysis starts by calculating FET-PET features, such as TBRmax, TBRmean, and/or TTP. These values are then fed either as single features or as a combination of more than one feature into a ROC analysis, resulting in a curve that characterizes the sensitivity and specificity as the decision threshold is altered [32]. It is important to recognize that the ROC curve visualizes the diagnostic accuracy for all possible decision thresholds.

**Figure 1:**
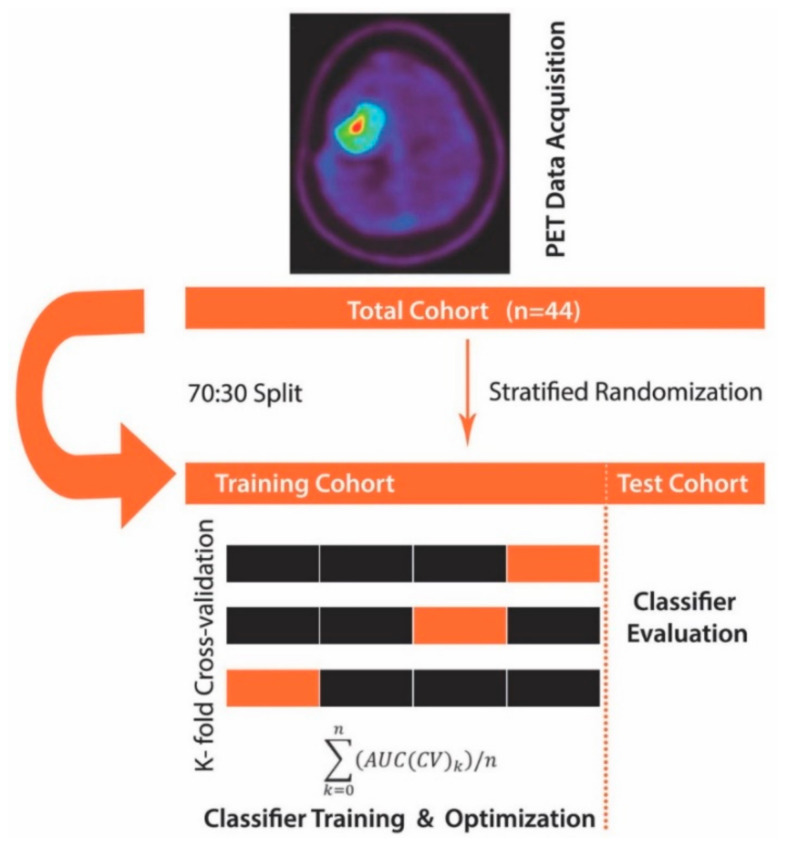
Machine learning algorithm schema. First, positron emission tomography (PET) data (the mean and maximum tumor-to-brain ratios (TBRmean, TBRmax) and time-to-peak (TTP)) were collected and fed to the machine learning algorithm, where the total cohort (*n* = 44) was subdivided into a training set and hold-out validation set (test) cohort in a 70:30 split. The training dataset underwent 3-fold cross-validation using a linear discriminant analysis (LDA) classifier. Finally, the so obtained and optimized classifier was evaluated for robustness and generalizability on the hold-out validation set cohort.

The approach used here differs in that several steps preceded the ROC analysis. First, we made use of linear discriminant analysis (LDA) with the least-squares solution solver and applied it to the calculated FET-PET features (TBRmax, TBRmean, and TTP). LDA allows maximizing the separability between two groups (here PSP and TP) by transforming the features into a lower-dimensional space. LDA is similar to principal component analysis (PCA), but it focuses on maximizing the separability between known groups. It is a supervised approach as opposed to PCA (unsupervised). Next, to allow for the least biased possible estimation of classification accuracy, we split our study cohort with 70% randomly allocated to the training set and 30% to the hold-out validation set while accounting for differences in the distribution of study groups (PSP, TP). The training cohort consisted of 30 patients, while the hold-out validation set cohort consisted of 14 patients (4 PSP; 10 TP). Notably, the hold-out validation set was left aside and only used once the model was trained, i.e., learned to correctly distinguish PSP from TP. Next, we proceeded with model training by using 3-fold stratified cross-validation to enhance classification performance (Figure 1. We deliberately opted for 3-fold stratified cross-validation given the dataset was imbalanced towards fewer PSP cases. In each training run, the LDA algorithm was applied to the PET features in the training folds and evaluated on the validation folds (Figure 1). The training model performance was determined by averaging the AUC across cross-validation folds. Finally, the overall model performance was evaluated on the hold-out validation dataset using ROC analysis that was fed with the LDA transformed features. The AUC metric then served to quantify classifier output quality, whereby an AUC close to 1.0 indicating a perfect model, whereas values closer to 0.5 reflecting a non-relevant model. To compare whether ROC curves differed significantly, we deployed a bootstrapping algorithm, wherein bootstrap replicates (*n* = 2000) were extracted from the data analyzed using the ML algorithm and data obtained through conventional FET-PET data analysis. For each replicate, the AUC difference was calculated and divided by the standard deviation of the bootstrap differences. The so obtained value was compared against the normal distribution. A relevant difference was defined when there was a significant deviation from normal distribution at an alpha level of 5%.

### 2.7. Statistical Analysis

A two-sided Student *t*-test was performed to compare the distribution of TBRs between groups. Optimal thresholds for TBRmax, TBRmean, and TTP were calculated using the Youden index. To measure the strength of association between the three input variables (TBRmax, TBRmean, TTP), we calculated the Pearson r correlation coefficient with numbers between 0.10 and 0.29 indicating a small association and numbers larger than 0.50 representing a substantial relationship. Python (version 3.7.1, Python Software Foundation) and R (version 3.5.3, The R Project for Statistical Computing) were used for statistical calculation and visualization.

### 2.8. Data Availability Statement

All published and from this study will be shared by request from a qualified investigator. 

## 3. Results

### 3.1. Patients’ Characteristics

**Figure 2:**
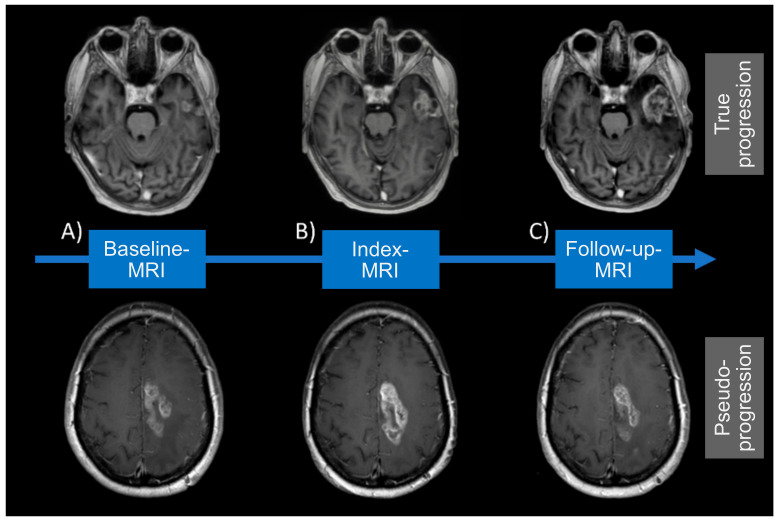
MRI visualization for true progression (TP) and pseudoprogression (PSP). MRI visualization for TP and PSP at (**A**) Baseline, (**B**) Index- and (**C**) Follow-up-MRI. True progression displays an increasing contrast-enhancement at Index- (**B**) and Follow-up MRI (**C**). Whereas pseudoprogression shows an enlarging contrast-enhancement at Index-MRI (**B**) but decreasing lesion on follow-up MRI (**C**).

On average, the index MRI, wherein PSP or TP was suspected, was conducted 11.4 weeks (mean value; range, 1.1–37.1 weeks) following the end of radiotherapy and the FET-PET 3.3 weeks later (mean value; range, 2.7–42.6 weeks). In patients who did not undergo surgery to confirm PSP/TP (77.3%), a follow-up MRI was obtained after a mean of 91.3 days and evaluated according to RANO criteria. The distribution of typical features in the study cohort in terms of age, KPS, the extent of resection, MGMT promoter methylation, and IDH1 mutation status was balanced between the TP (*n* = 30) and PSP (*n* = 14) group (Table 1). There was, however, a significant gender imbalance with the fraction of female patients being higher in the PSP cohort as compared to TP. The chemotherapy regimens used (TMZ or TMZ/CCNU) were equally distributed between groups (Table 1). A patient-wise presentation of study cohort characteristics is provided in Table S1. Representative examples of TP and PSP are visualized in Figure 2. 

### 3.2. Distribution of Conventional PET Features between PSP and TP

**Figure 3:**
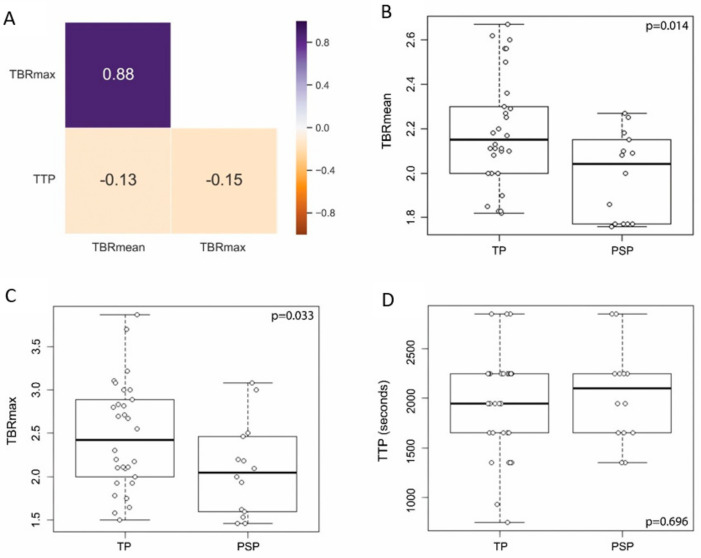
Correlation and distribution of PET derived features. (**A**) TBRmax and TBRmean showed a high Pearson’s correlation coefficient (0.88), whereas TTP did neither correlate with TBRmax (−0.15) nor TBRmean (−0.13). Boxplots with overlaid dot plots (**B**) showing significantly lower TBRmean in the PSP group (mean ± standard deviation (sd), 1.99 ± 0.20) compared to the TP group (mean ± sd, 2.19 ± 0.25) (*p* = 0.014, Student’s *t*-test), (**C**) significantly lower TBRmax in the PSP group (mean ± sd, 2.08 ± 0.54) compared to the TP group (mean ± sd, 2.45 ± 0.63) (*p* = 0.033, Student’s *t*-test), and (**D**) no significant difference in TTP between PSP (mean ± sd, 2036 ± 477) and TP (mean ± sd, 1926 ± 518) (*p* = 0.696, Student’s *t*-test).

The PET diagnostic outputs several features that have been found to be important. The most widely used are TBRmax, TBRmean, and TTP. In a correlation analysis using the Pearson r coefficient TBRmax and TBRmean had a high relationship (*r* = 0.88), whereas TTP was not associated with TBRmax or TBRmean (*r* = −0.15 and *r* = −0.13, respectively) (Figure 3A) indicating a putative additional benefit from including TTP data to the analysis. The distribution of data points between TP and PSP was investigated and compared revealing a significant difference between those groups for TBRmax (*p* = 0.033) and TBRmean (*p* = 0.014) with lower values pertaining to the PSP group. TTP was not different between PSP and TP (*p* = 0.696, Figure 3B–D).

### 3.3. PSP Detection Applying Conventional PET Analysis

**Figure 4:**
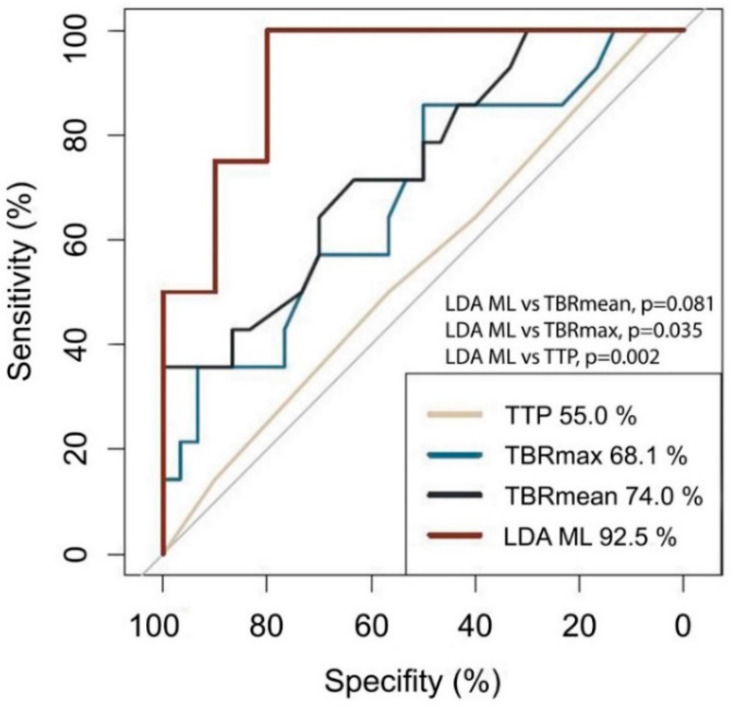
ROC plot. The AUC is lowest for TTP (55.0%), followed by TBRmax (68.1%) and TBRmean (74.0%). The highest AUC is reached using the LDA ML model (92.5%) and is significantly different from that of TBRmax (*p* = 0.035) and TTP (*p* = 0.002).

To determine whether any of the standard features can detect PSP accurately (TBRmax, TBRmean, TTP), we estimated the AUC metric for each of them. As shown in Figure 4, TTP had the worst classification performance corresponding to an AUC of 55% (95% Confidence interval [CI], 37–73%). TBRmax and TBRmean had a somewhat better AUC with values of 68% (95% CI, 51–86%) and 74% (95% CI, 58–90%), respectively. Furthermore, we investigated whether the combination of TBRmax, TBRmean, or TTP may provide improved AUC values. For this purpose, we defined the optimal cutoffs for TBRmax, TBRmean and TTP, being 2.55 (sensitivity 50.0%, specificity 85.7%), 1.82 (sensitivity 100.0%, specificity 35.7%), and 12.5 minutes (sensitivity 100.0%, specificity 0.0%), respectively. Based on these cutoffs, the three features were combined using the logical OR operator and resulted in a slightly increased AUC of 77% (95% CI, 63–92%). To determine whether MGMT gene promoter methylation status is of value for discriminating PSP from TP we ran a ROC analysis using MGMT gene promoter methylation status as the only predictor. The results with AUC of 63.6 (95% CI, 47.8–79.4%) indicate that MGMT gene promoter methylation status alone does not lend itself to predict PSP/TP. 

### 3.4. Implementation of The Machine Learning Algorithm

Using an ML model based on the LDA approach, the AUC could be improved up to 93% (95% CI, 78–100%; sensitivity 100%; specificity 80%), indicating a good classification ability (Figure 3). The AUC in the training dataset was equally high, with an AUC of 95% indicating a low propensity for overfitting. Using a bootstrapping method, we compared the AUC for LDA ML with that of TBRmax and TBRmean and a combination of TBRmax, TBRmean, and TTP, showing a significant difference compared to TBRmean (*p* = 0.035) and a difference with a non-significant *p*-value for TBRmax (*p* = 0.081) and the combination of TBRmax, TBRmean, and TTP (*p* = 0.132).

## 4. Discussion

Our results indicate that ML-enhanced FET-PET analysis—compared to conventional FET-PET ROC analysis—may allow for better differentiation between pseudoprogression and true tumor progression in IDH-wildtype glioblastoma patients.

Compared to our previous study [15], the AUC values for TBRmax and TBRmean are formally slightly different from those in the current study. This could be explained by the fact that the current study included GBM patients with wildtype IDH only, thus referring to a different population. To our knowledge, among the articles published about PSP detection using FET-PET in IDH-wildtype glioblastoma, our results provide the highest level of diagnostic accuracy [7,15,33]. It should be noted, however, that this study is the first to investigate the capacity of FET-PET based PSP detection exclusively in glioblastoma with IDH-wildtype status. The reason for restricting our analysis to IDH-wildtype glioblastoma roots in the substantial changes made in the most recent World Health Organization (WHO) classification for central nervous system tumors, wherein IDH-wildtype and IDH-mutant glioblastoma have been defined as separate tumor entities given their contrasting clinical outcome [17]. It should be alerted to the fact that IDH testing in patients younger than 55 years could reveal false negative results, when relying on immunohistochemistry only [34]. In this study, we accounted for that by performing DNA sequencing in patients older than 55 years to capture all possible IDH variants, including those not assessed by immunohistochemistry. Interestingly, as has been shown in a recent study, the diagnostic accuracy to differentiate TP from PSP in WHO grade II-IV glioma may be dependent upon IDH mutation status with higher accuracies observed in the IDH-wildtype glioma group [16]. We noticed that the accuracies obtained for TBRmax, TBRmean and TTP at optimal cutoffs, as detected through the Youden’s index, were formally lower that those reported in previous studies [7,31]. The most apparent explanation could be related to the different cohort under investigation in this study, namely IDH wildtype glioblastoma exclusively, indicating a different biology. In our study, the rate of pseudoprogression in patients with methylated MGMT promoter as reported in other studies [35,36] could not be observed, which might be related to the lower number of patients in the present data set. The overlap of TBR values between PSP and TP limits the achievable accuracy in distinguishing PSP and TP and highlights the need for advanced image analysis, as presented in our study, or by combining FET-PET with other modalities, such as perfusion MRI or MRI spectroscopy, to further increase diagnostic accuracy. Apart from FET-PET, advanced MR techniques, also introducing ML, have been shown to be useful in the non-invasive diagnosis of pseudoprogression. One study addressed the value of ML-enhanced exploration of radiomic features derived from MR perfusion scans and reported AUC values ranging from 89% to 94% [35]. Notably, the authors decided to include IDH-mutant and IDH-wildtype glioblastoma into the analysis, imparting uncertainty on how clinically relevant the results are. MR perfusion-weighted MRI (PWI) might be an alternative approach to differentiate early tumor progression from pseudoprogression. However, although consensus recommendations for an improved standardization of the dynamic susceptibility contrast PWI protocol have been recently published [36], the comparability of the results from PWI are hampered by the variable levels of standardization across many centers in terms of data acquisition and post-processing [37]. In contrast, PET guidelines for data acquisition, reconstruction, and post-processing are available and widely implemented and allow for a better comparability of results between different centers [26]. Another practical reason for the application of FET PET in our study is the fact that PWI is neither established in our institution nor reimbursed by the insurance companies, whereas the latter two apply to FET PET. Moving forward, it would be tempting to investigate whether joint information from MR and PET data would increase the PSP discrimination performance using an ML approach.

Our results show the potential of machine learning-based algorithms compared to conventional FET-PET data analysis in PSP detection in IDH-wildtype glioblastoma. The LDA classifier was significantly better than TBRmean, but not better than TBRmax. One possible explanation could be that our sample size is too low as to capture a possible difference between TBRmax and LDA, too. The machine learning approach is only useful, however, when overfitting is accounted for. Overfitting holds true when an ML model tends to overfit to the training dataset and, therefore, may not lend itself to be used on an independent dataset. It can be diagnosed by investigating the accuracy in the training set and comparing it to the hold-out validation set [38]. In the current study, the AUC in the training dataset was very similar (AUC, 95%) to that in the hold-out validation dataset (AUC, 92.5%), indicating that overfitting is unlikely. It should be mentioned, however, that the overall sample size is rather low (*n* = 44) to provide a robust ML model, ready to be used in clinical practice. A larger sample size and deployment of more powerful ML models, such as deep learning based, would be desirable for future studies in order to build a robust ML model.

We noticed that there was a preponderance of female patients in our PSP cohort. This contrasts with other glioblastoma PSP cohorts that have been published in the past [15]. The major difference of our cohort to the published ones is that we restricted to the IDH wt glioblastoma entity, possibly accounting for the observed difference. More research is needed to understand the association of IDH mutation status and pseudoprogression. 

Possible limitations of this study are the retrospective nature of the analysis as well as the relatively small sample size, even though decent compared to other studies [7,10,15]. Furthermore, the data was skewed in a way that PSP was less frequent than TP, which is not uncommon in this type of study given the low prevalence of PSP. In fact, skewed datasets are pretty much the standard in studies investigating pseudoprogression [7,10,16,35]. This imbalance of classes should be considered when interpreting accuracy. To cope with this problem of skewed data, pursuant to published recommendations for skewed datasets, we have chosen AUC—instead of accuracy—as the target metric as it is less prone to data imbalance [39]. Besides, the contribution of different chemotherapy regimens, particularly temozolomide as opposed to temozolomide plus lomustine [40], may have varying impact on the frequency and appearance of pseudoprogression in IDH-wildtype glioblastoma. Moreover, it should be mentioned that the diagnosis of PSP was confirmed based on the histopathological analysis in only 22% of cases leaving a certain degree of uncertainty regarding the final diagnosis. It should be noted, that given the risks associated with surgery, obtaining a biopsy for the sole purpose of differentiating PSP from TP may not be reasonable in many cases as even with histopathological analysis, it may be difficult to ascertain pseudoprogression with confidence.

In conclusion, this retrospective analysis furnishes an example of the potential of ML in the non-invasive FET-PET-based diagnosis of pseudoprogression in patients with IDH-wildtype glioblastoma. To confirm the reliability of this method, confirmation in a larger multi-institutional cohort, the introduction of more advanced machine learning algorithms and the inclusion of other imaging modalities are desirable.

## 5. Conclusions

This preliminary study indicates that machine learning enhanced FET-PET may provide improved accuracy as compared to conventional FET-PET. However, further studies with a higher sample size are needed to confirm the results.

## Figures and Tables

**Table 1:** Patients’ characteristics.

Characteristic	True Progression (*n* = 30)	Pseudo-Progression (*n* = 14)
Tumor entity, n (%)		
Primary IDH-wildtype glioblastoma	30 (100)	14 (100)
Gender, n (%)		
Male	28 (93)	6 (43)
Female	2 (7)	8 (57)
Age at diagnosis [y], median (range)	59 (42–79)	51 (34–76)
KPS at the time of index MRI, median (range)	85 (60–100)	90 (70–100)
Extent of resection, n (%)		
Complete resection	14 (47)	5 (36)
Partial resection	11 (37)	4 (29)
Biopsy	5 (17)	5 (36)
Confirmation by histopathology, n (%)		
Yes	7 (23)	3 (21)
No	23 (77)	11 (79)
MGMT promotor methylation, n (%)		
No	20 (67)	5 (36)
Yes	9 (30)	8 (57)
Missing	1 (3)	1 (7)
Prior treatment, n (%)		
Radiotherapy	30 (100)	14 (100)
TMZ	30 (100)	14 (100)
CCNU	3 (10)	1 (7)
No chemotherapy	0 (0)	0 (0)
Concomitant dexamethasone treatment, n (%)		
No	20 (67)	9 (64)
Yes	9 (30)	5 (36)
Missing	1 (3)	0 (0)
Change in dexamethasone dose between index and follow-up MRI, n (%)		
No	8 (27)	5 (36)
Yes	21 (70)	9 (64)
Missing	1 (3)	0 (0)
FET PET features		
TBR_mean_, median (range)	2.15 (1.82–2.67)	2.04 (1.76–2.27)
TBR_max_, median (range)	2.52 (1.82–3.87)	2.09 (1.76–3.08)
TTP [min], median (range)	32.5 (12.5–47.5)	35 (22.5–47.5)

Abbreviations: CCNU, lomustine; DNA, Deoxyribonucleic acid; FET, O-(2-[18F]-fluoroethyl)-L-tyrosine; IDH, isocitrate dehydrogenase; KPS, Karnofsky Performance Status; MGMT, O-6-methylguanine-DNA methyltransferase; MIN, Minutes; MRI, Magnetic Resonance Imaging; PET, positron emission tomography; TMZ, temozolomide.

## Supplementary Materials

The following are available online at https://www.mdpi.com/2072-6694/12/11/3080/s1, Table S1: Patient-wise study cohort details.
