# Supplementary material for: A Preliminary Study on Machine Learning-Based Evaluation of Static and Dynamic FET-PET for the Detection of Pseudoprogression in Patients with IDH-Wildtype Glioblastoma
Source: Cancers (Basel). 2020 Oct 22;12(11):3080. doi: 10.3390/cancers12113080 (PMC7690380; doi:10.3390/cancers12113080)
Supplement: Supplementary file 1 [file cancers-12-03080-s001.pdf]

Supplementary Materials

A Preliminary Study on Machine Learning-Based Evaluation of Static and Dynamic FET-PET for The Detection of Pseudoprogression in Patients with IDH-Wildtype Glioblastoma

Sied Kebir, Teresa Schmidt, Matthias Weber, Lazaros Lazaridis, Norbert Galldiks, Karl-Josef Langen, Christoph Kleinschnitz, Elke Hattingen, Ulrich Herrlinger, Philipp Lohmann and Martin Glas

Table S1. Patient-wise study cohort details.

No°	Gender	Age at Diagnosis [y]	KPS at The Time of Index MRI	Histological Diagnosis	MGMT Promoter Methylation	IDH1/2 Mutation	Line of Therapy	Extent of Resection	Therapy Regimen from Tumor Diagnosis until Index MRI/ PET Investigation	Concomitant Dexamethasone Treatment	Change in Dexamethasone Dose between Index- and Follow-up MRI	Follow-up Diagnosis	Histological Confirmation of Follow-up Diagnosis	TBRmean	TBRmax	TTP [min]
1	m	46	90	GBM	yes	no	1st	CR	P:cR,RT+CCNU+TMZ,2ZCCNU+TMZ	4 mg	−2 mg	PSP	no	2.25	3.08	37.5
2	f	52	70	GBM	no	no	1st	B	P:B,RT+TMZ	8 mg	−4 mg	PSP	no	1.77	1.77	22.5
3	m	46	70	GBM	yes	no	1st	B	P:pR,RT+TMZ,6ZTMZ	no	no	PSP	no	2.08	2.5	22.5
4	m	50	80	GBM	yes	no	1st	B	P:B,RT+TMZ,3ZTMZ/CCNU	5.5 mg	+6.5 mg	PSP	no	1.86	1.93	27.5
5	m	59	90	GBM	no	no	1st	CR	P:cR,RT+TMZ	no	no	TP	no	2.56	3.11	32.5
6	m	58	90	GBM	yes	no	1st	PR	P:pR,RT+TMZ	n.a.	n.a.	TP	no	2	2	47.5
7	f	44	90	GBM	no	no	1st	CR	P=cR,RT+TMZ,2ZTMZ	no	no	TP	no	2.3	2.7	37.5
8	m	60	90	GBM	no	no	1st	CR	P:cR,RT+TMZ,5ZTMZ	4 mg	no	TP	no	2.62	3	22.5
9	m	66	90	GBM	no	no	1st	CR	P:cR,RT+TMZ,4ZTMZ	no	no	TP	no	2.5	2.5	37.5
10	m	64	80	GBM	yes	no	1st	PR	P:pR,RT+TMZ,CCNU+TMZ	30 mg	no	TP	no	1.82	1.82	37.5
11	m	59	80	GBM	no	no	1st	PR	P:pR,RT+TMZ	no	no	TP	no	2	2.09	32.5
12	m	61	100	GBM	no	no	1st	CR	P.cR,RT+TMZ,5ZTMZ	no	+24 mg	TP	no	1.83	1.83	15.5

13	m	74	90	GBM	no	no	1st	B	P:B,RT+TMZ	1.5 mg	−0.5 mg	TP	no	2.56	3.22	37.5
14	m	50	90	GBM	yes	no	1st	PR	P:R,RT+TMZ+CCNU,5ZTMZ+CCNU	no	no	TP	no	2	2.11	47.5
15	m	61	90	GBM	no	no	1st	CR	P:cR,RT+TMZ,6ZTMZ	no	no	TP	no	2.11	2.11	37.5
16	m	74	80	GBM	yes	no	1st	PR	P:R,RT+TMZ,2ZTMZ	no	no	TP	no	2.29	2.71	37.5
17	m	58	70	GBM	no	no	1st	CR	P:cR,RT+TMZ,4ZTMZ	8 mg	4 mg	TP	no	2.18	2.55	27.5
18	m	70	90	GBM	no	no	1st	PR	P:pR,RT+TMZ,6ZTMZ	no	no	TP	no	2.36	3	22.5
19	m	48	90	GBM	no	no	1st	CR	P:cR+RT+TMZ,1ZTMZ	no	no	TP	yes	2.11	2.11	27.5
20	m	57	80	GBM	yes	no	1st	CR	P:cR,RT+CCNU+TMZ,3ZCCNU+TMZ	4 mg	−2 mg	TP	yes	1.9	1.9	37.5
21	f	76	80	GBM	yes	no	1st	CR	EORTC/NCIC 26981	6 mg	−4 mg	PSP	yes	2.18	2.18	37.5
22	f	58	90	GBM	no	no	1st	PR	EORTC/NCIC 26981	no	no	PSP	no	2	2.09	32.5
23	m	50	100	GBM	no	no	1st	CR	EORTC/NCIC 26981	no	no	PSP	no	2.09	2.09	37.5
24	m	34	80	GBM	yes	no	1st	PR	EORTC/NCIC 26981	no	no	PSP	no	1.77	1.77	37.5
25	f	48	100	GBM	n.a.	no	1st	CR	EORTC/NCIC 26981	no	no	PSP	yes	1.77	1.77	27.5
26	f	64	100	GBM	yes	no	1st	CR	EORTC/NCIC 26981	no	no	PSP	no	1.77	1.77	47.5
27	f	66	80	GBM	no	no	1st	B	EORTC/NCIC 26981	no	no	PSP	no	2.27	3	47.5
28	m	66	100	GBM	yes	no	1st	B	EORTC/NCIC 26981	no	no	PSP	no	2.15	2.46	27.5
29	f	49	90	GBM	no	no	1st	PR	EORTC/NCIC 26981	8mg	−6 mg	PSP	no	2.1	2.2	37.5
30	f	72	100	GBM	yes	no	1st	PR	EORTC/NCIC 26981	no	no	PSP	yes	1.76	1.76	32.5
31	m	61	80	GBM	no	no	1st	CR	EORTC/NCIC 26981	no	no	TP	yes	2.2	2.2	37.5
32	m	52	80	GBM	no	no	1st	PR	EORTC/NCIC 26981	no	no	TP	yes	2.27	2.82	32.5
33	m	69	80	GBM	no	no	1st	PR	EORTC/NCIC 26981	no	no	TP	yes	1.9	1.9	37.5
34	m	59	80	GBM	no	no	1st	PR	EORTC/NCIC 26981	no	no	TP	yes	1.85	1.92	37.5
35	m	52	100	GBM	no	no	1st	CR	EORTC/NCIC 26981	no	no	TP	no	2.1	2.3	32.5
36	m	72	70	GBM	no	no	1st	B	EORTC/NCIC 26981	no	no	TP	no	2.6	3.7	32.5
37	m	51	100	GBM	yes	no	1st	PR	EORTC/NCIC 26981	4 mg	−4 mg	TP	yes	2.67	3.87	12.5
38	m	44	80	GBM	no	no	1st	CR	EORTC/NCIC 26981	no	no	TP	no	2.11	2.89	47.5
39	m	61	60	GBM	no	no	1st	B	EORTC/NCIC 26981	10 mg	−6 mg	TP	no	2.25	3.08	27.5
40	m	57	70	GBM	no	no	1st	B	EORTC/NCIC 26981	2 mg	−2 mg	TP	no	2.13	2.8	22.5
41	m	42	100	GBM	yes	no	1st	CR	EORTC/NCIC 26981	no	no	TP	no	2.17	2.67	37.5
42	m	79	100	GBM	n.a.	no	1st	CR	EORTC/NCIC 26981	no	no	TP	yes	1.83	1.83	27.5
43	m	76	80	GBM	yes	no	1st	B	EORTC/NCIC 26981	no	no	TP	no	2.25	2.83	27.5
44	f	52	60	GBM	yes	no	1st	PR	EORTC/NCIC 26981	3 mg	−3 mg	TP	no	2.08	2.08	22.5

Abbreviations: B, biopsy; CCNU, lomustine; CR, complete resection; EORTC/NCIC 26981, treatment according to Stupp's protocol; f, female; GBM, glioblastoma; IDH, isocitrate dehydrogenase; KPS, Karnofsky Performance Status; m, male; MGMT, O-6-methylguanine-DNA methyltransferase; min, minutes; MRI, Magnetic Resonance Imaging; No°, number; PET, positron emission tomography; PR, partial resection; PSP, pseudoprogression; RT, radiotherapy; TMZ, temozolomide; TP, tumor progression; y, years.

© 2020 by the authors. Licensee MDPI, Basel, Switzerland. This article is an open access article distributed under the terms and conditions of the Creative Commons Attribution (CC BY) license (http://creativecommons.org/licenses/by/4.0/).
